# Supplementary material for: Impact of Androgen Deprivation Therapy Associated to Conformal Radiotherapy in the Treatment of D’Amico Intermediate-/High-Risk Prostate Cancer in Older Patients
Source: Cancers (Basel). 2020 Dec 29;13(1):75. doi: 10.3390/cancers13010075 (PMC7795189; doi:10.3390/cancers13010075)
Supplement: Supplementary file 1 [file cancers-13-00075-s001.pdf]

Table S1: Adverse events linked to Androgen Deprivation Therapy (ADT)

Symptoms	N	(%)
**General symptoms**	9	(9.0)
Asthenia grade ≥2	7	(6.9)
Weight gain ≥5%	2	(1.9)
**Locomotor events**	17	(16.8)
Arthralgia grade ≥2	2	(1.9)
Myositis grade ≥2	1	(0.9)
Osteoporotic fracture	4	(3.9)
**Cardiovascular events**	23	(22.8)
Coronaropathy	6	(5.9)
Cerebrovascular disease	3	(2.9)
Congestive heart failure	4	(3.9)
Phlebitis or pulmonary embolism	2	(1.9)
Carotide stenosis	3	(2.9)
**Neurological events**	7	(6.9)
Cognitive disorders	7	(6.9)
**Biological events**	3	(2.9)
Anemia (no cause)	2	(1.9)
Hepatic cytolysis grade ≥2	1	(0.9)
**Total**	44	(43.6)
**Stop ADT due to AE**	10	(10.0)
**Death due to AE**	2	(1.9)

AE ; Adverse Events ; ADT : Androgen Deprivation Therapy ; N : Number

Table S2: a) Cardiovascular events in older patients (groups A + B ; N = 101). Univariate analysis.

Variables	OR	CI 95%	p-value
**Age (≥82 vs <82 years)**	**5.57**	**1.42-21.86**	**0.014**
Intermediate-risk (IR)/ high-risk (HR)	1.49	0.43-5.19	0.528
ADT median duration (≤6 vs >6 months)	1.83	0.52-6.43	0.344
Karnofsky score (≥100% vs <100%)	0.63	0.09-4.24	0.635
Charslon score (≤5 vs >5)	2.40	0.61-9.49	0.212
ACE 27 score (≤1 vs >1)	0.88	0.18-4.17	0.867
Anticoagulant and/or antiaggregant	2.92	0.78-10.92	0.112
Antidiabetes drug	1.25	0.24-6.54	0.792
Antihypertensive drug	1.91	0.48-7.64	0.361
Normalization of testosterone levels	0.82	0.24-2.84	0.752

b) Cardiovascular events in older patients (groups A + B; N = 101). Multivariate analysis.

Variables	OR	CI 95%	p-value
**Age (≥82 vs <82 years)**	**7.15**	**1.52-33.75**	**0.013**
Charslon score (≤5 vs >5)	1.26	0.24-6.55	0.783
Anticoagulant and/or antiaggregant	3.54	0.67-18.81	0.138

ADT : Androgen Deprivation Therapy ; HR : High-risk, IR : Intermediate-risk; N: Number**;** **OR** : Odd Ratio ; **CI**: Confidence Interval
